# Supplementary figures and images for: Controlling periodic long-range signalling to drive a morphogenetic transition
Source: eLife. 2023 Mar 1;12:e83796. doi: 10.7554/eLife.83796 (PMC10027319; doi:10.7554/eLife.83796)

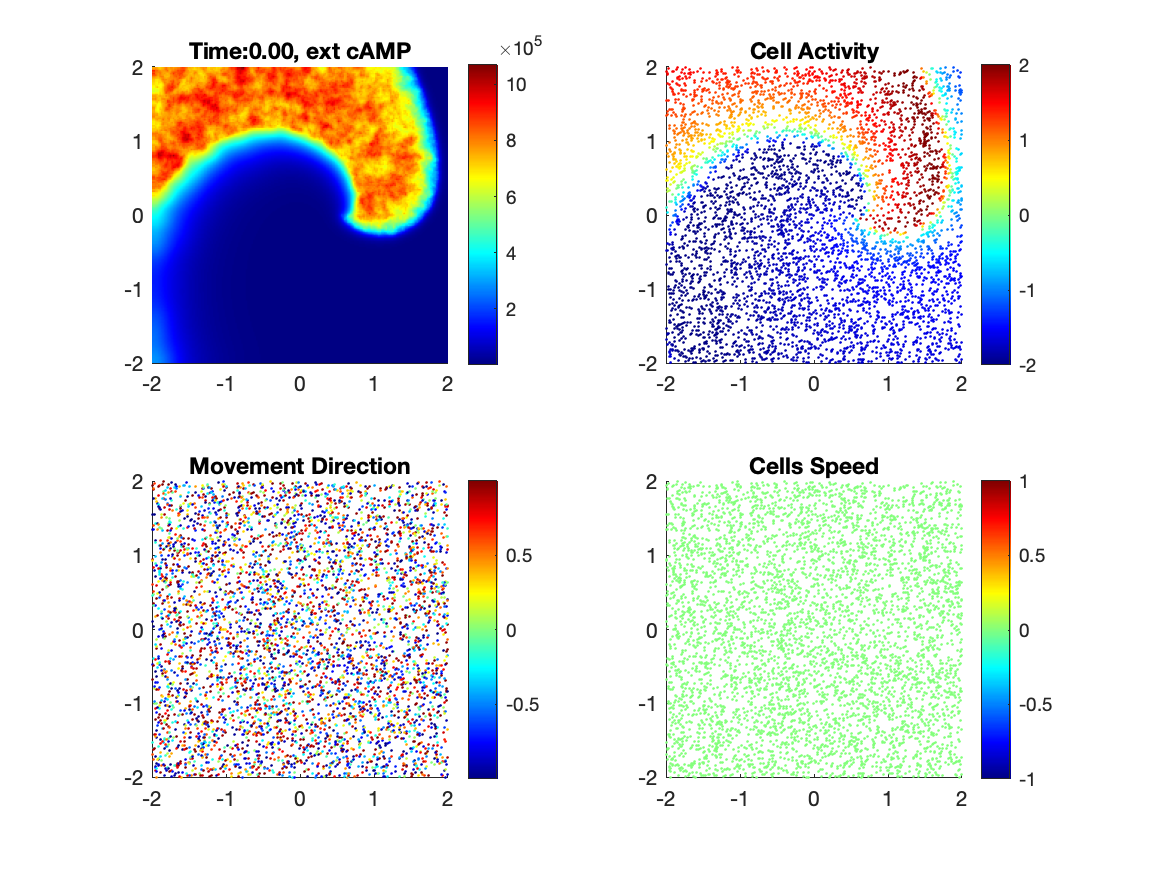

Supplement: Source code 1. [file elife-83796-code1.zip › Code_Paper_Version/Results/14Jan2022_11_31_26/pic_0.png]

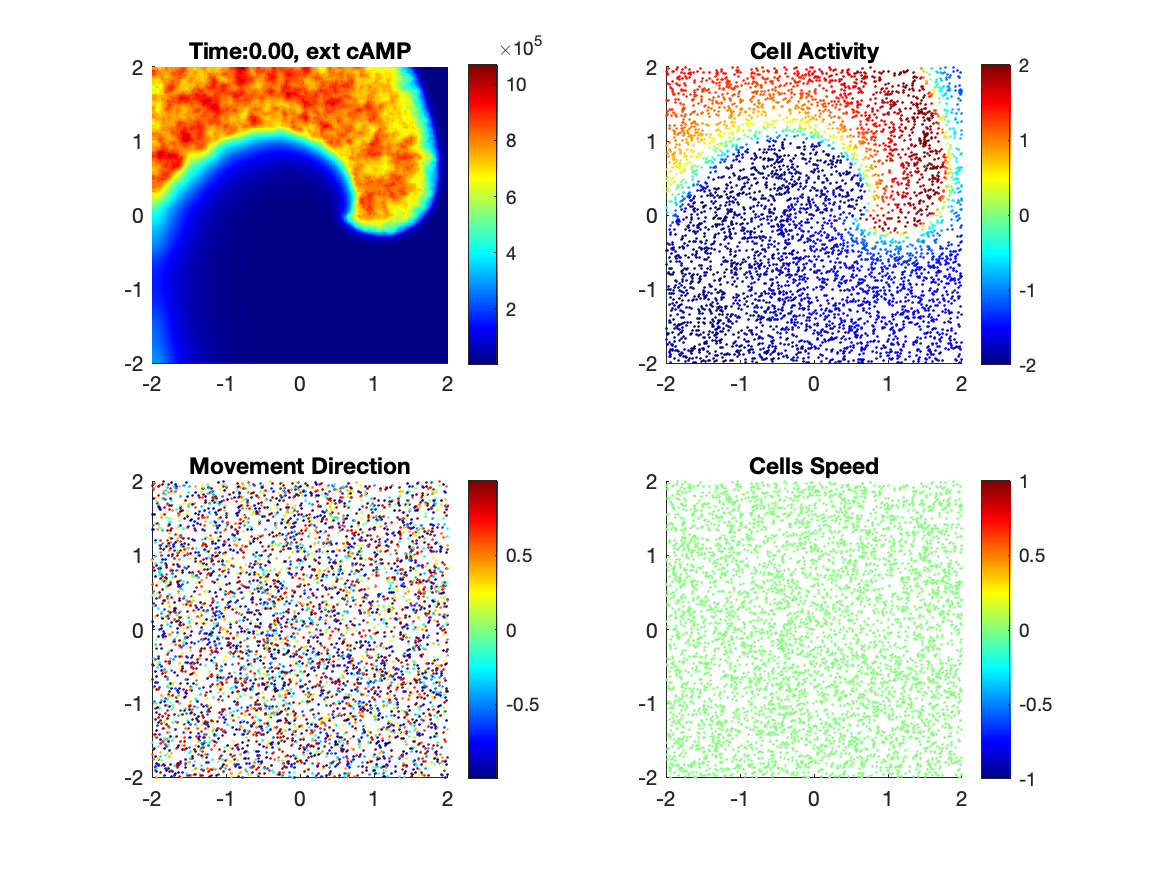

Supplement: Source code 1. [file elife-83796-code1.zip › Code_Paper_Version/Results/14Jan2022_11_39_22/pic_0.png]

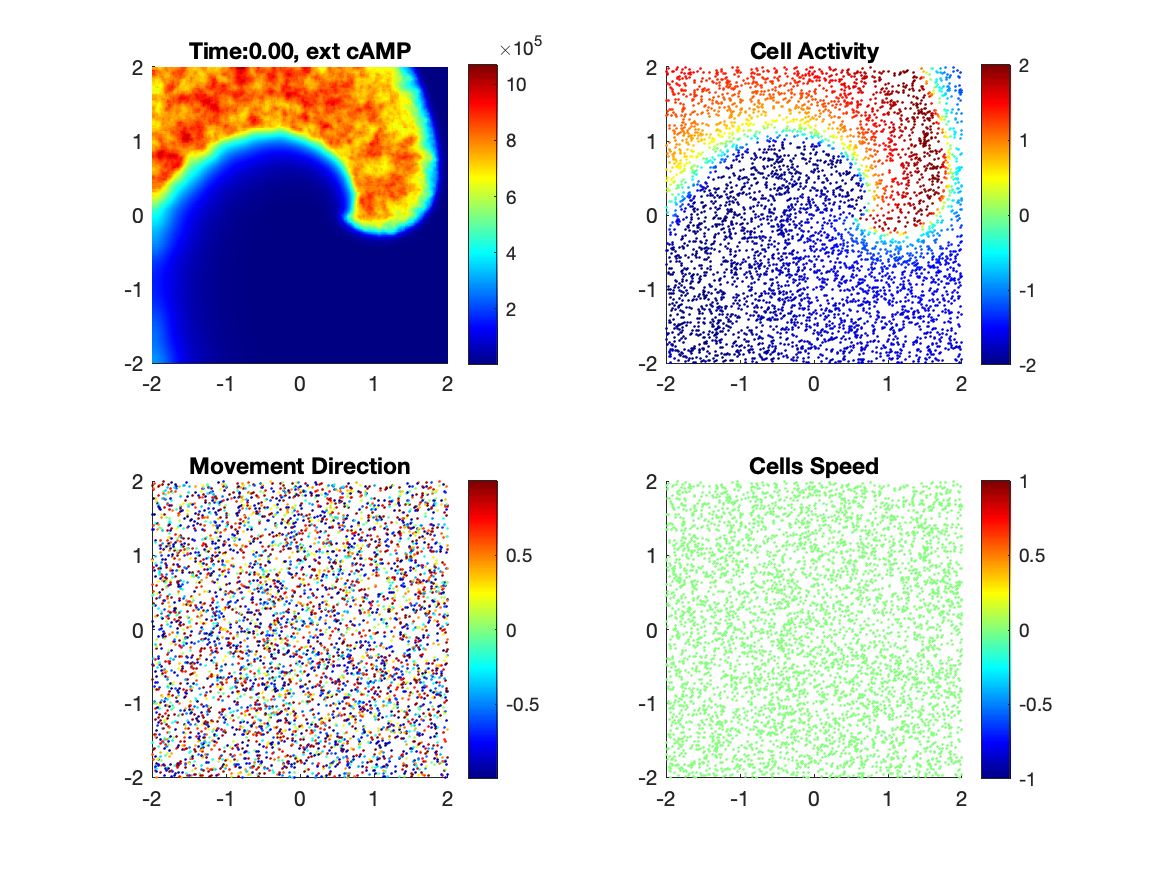

Supplement: Source code 1. [file elife-83796-code1.zip › Code_Paper_Version/Results/14Jan2022_13_36_05/pic_0.png]

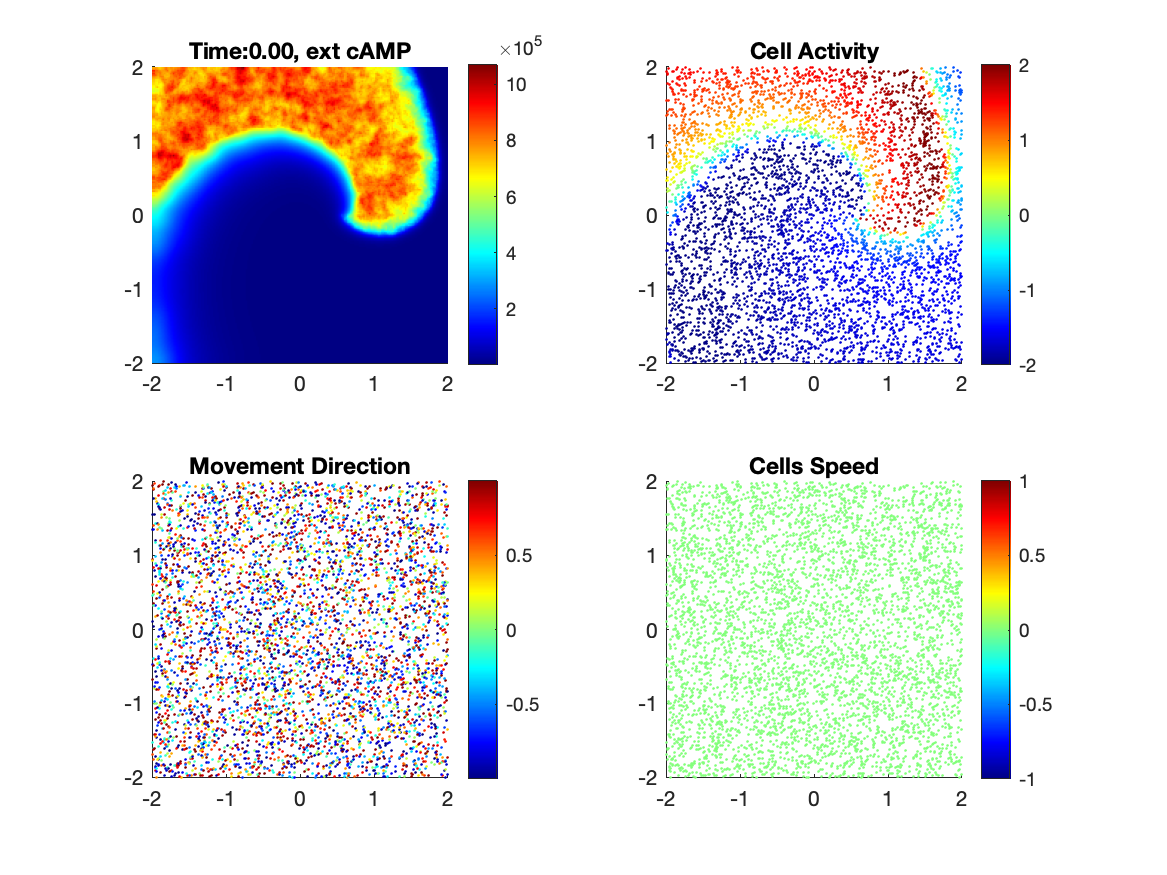

Supplement: Source code 1. [file elife-83796-code1.zip › Code_Paper_Version/Results/14Jan2022_12_27_19/pic_0.png]

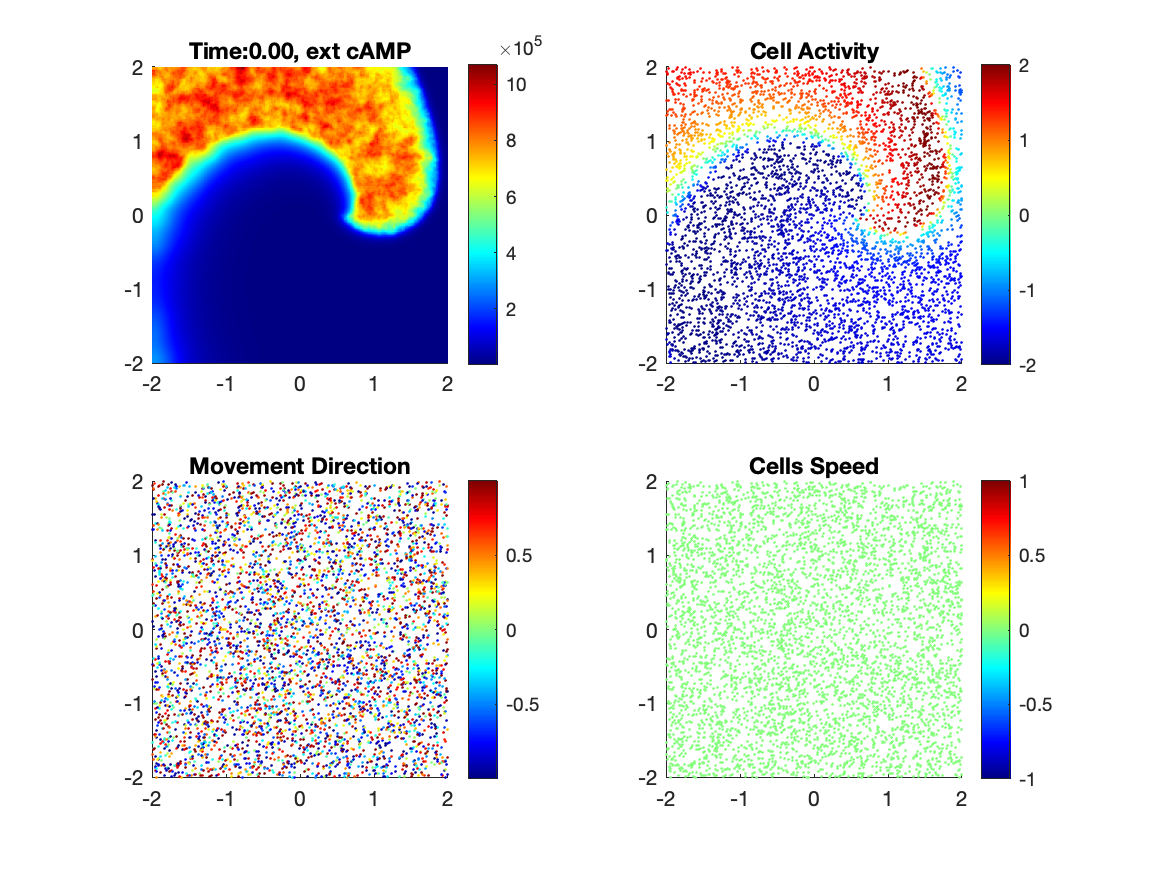

Supplement: Source code 1. [file elife-83796-code1.zip › Code_Paper_Version/Results/14Jan2022_11_31_46/pic_0.png]

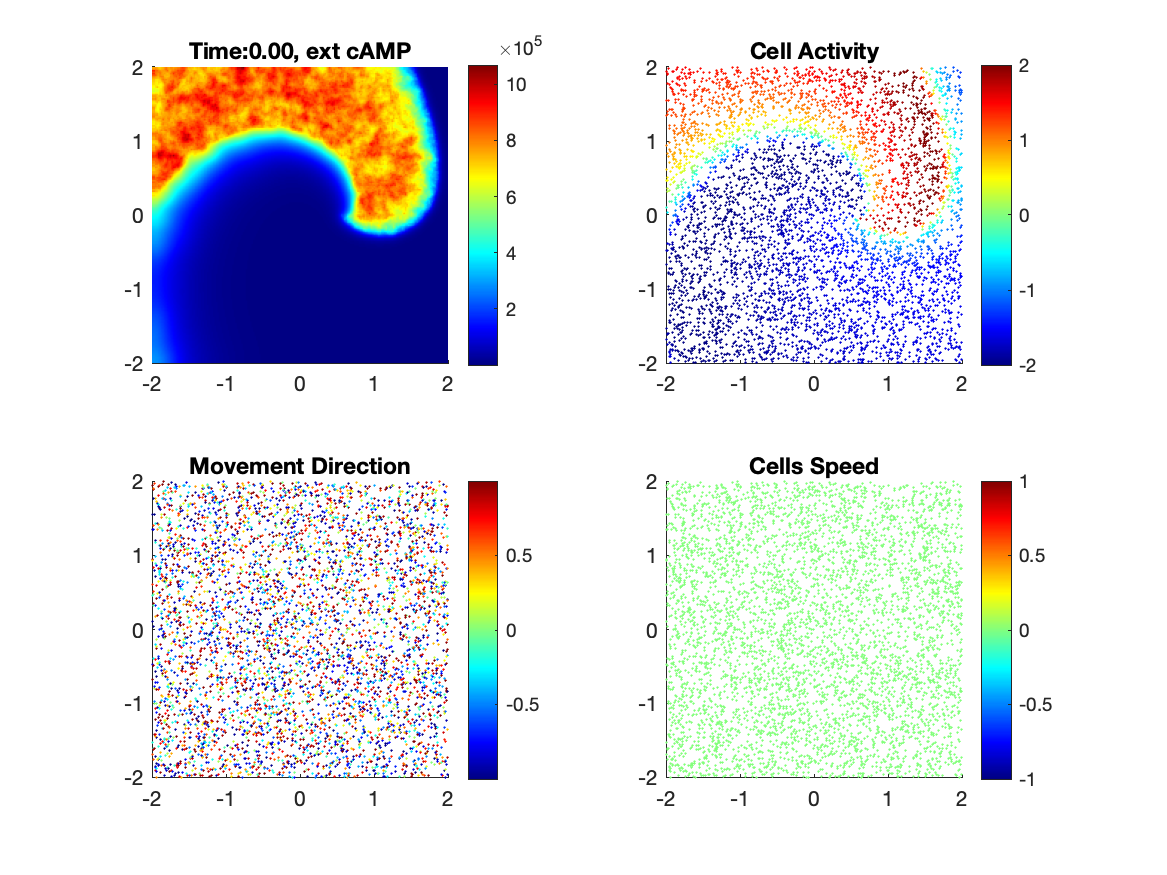

Supplement: Source code 1. [file elife-83796-code1.zip › Code_Paper_Version/Results/14Jan2022_18_23_47/pic_0.png]

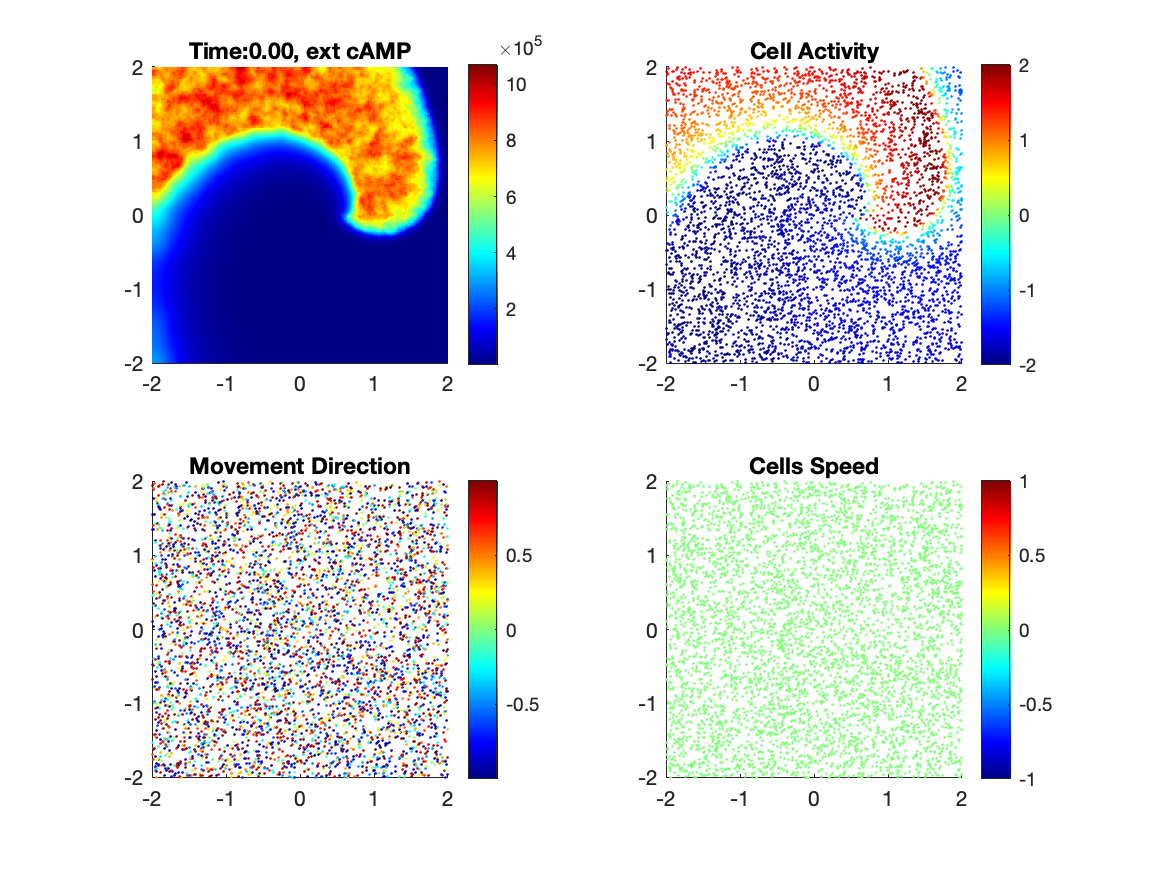

Supplement: Source code 1. [file elife-83796-code1.zip › Code_Paper_Version/Results/14Jan2022_13_36_20/pic_0.png]
